# Building the injury field in North America: the perspective of some of the pioneers

**DOI:** 10.1186/s40621-018-0177-4

**Published:** 2018-12-21

**Authors:** David Hemenway

**Affiliations:** Harvard TH Chan School of Public Health, 677 Huntington Avenue, Boston, MA 02115 USA

**Keywords:** Injury field, Injury pioneers, History

## Abstract

**Background:**

After the publication in 1985 of *Injury in America* and the establishment of an injury center at the Centers for Disease Control, there was a concerted attempt to create an “injury field.”

**Main body:**

Thirty-six (36) pioneers in the injury prevention field responded to questions about the major accomplishments and failures of their profession since the publication of the seminal Institute of Medicine report *Injury in America* in 1985. Much has been accomplished. Indeed, it is difficult to believe that before the 1990s there was no federal agency focused on preventing fall injuries, drownings, sport concussions or bullying in schools. There was no readily available surveillance data on fatal injuries, no national associations of injury researchers or practitioners, no American Public Health Association (APHA) injury and emergency health services (ICEHS) section and few injury journals. Hardly anyone wore seatbelts and virtually no cigarettes were fire-safe. Sadly, there has been little success at limiting firearm and overdose deaths as injury prevention remains a step-child in the health field with funding not nearly commensurate to the size of the problem. Training in effective advocacy has been proposed both to help attract funding and reduce injuries.

**Conclusion:**

Injury prevention pioneers have much to teach current public health students, researchers and practitioners about the history and future of the field.

## Background

After the publication in 1985 of *Injury in America* (National Research Council, 1985) and the establishment of an injury center at the Centers for Disease Control (CDC), there was a concerted attempt to create an “injury field.” Since the first generation of injury researchers and practitioners are retiring or retired (and before too many die) it seemed important to gather some of the collective wisdom of these early pioneers.

At the beginning of 2018, I sent an email with four questions to fifty-eight eminent injury researchers and practitioners in the United States (and one in Canada) who had been around at the creation of the field. Over the years, these professionals provided injury leadership at the local, state and national level, contributed substantially to the peer reviewed literature, provided education and mentorship to innumerable students, developed sustainable community outreach programs and engaged in local, state and national policy efforts to reduce the injury burden on society. Thirty-six were kind enough to respond (62% response rate). Here is a summary of their responses.

The first question asked, “Since the mid-1980s, what do you see as 1–3 of the major accomplishments of the injury field?” A second question asked about major disappointments for the field, a third question asked about things the respondent was personally proud of in terms of helping to build the injury field and a final question solicited advice for young injury researchers and practitioners. Here I report on responses to the first, second and fourth questions.

In terms of accomplishments, three themes emerged from the responses: (1) creating the systems and institutions needed for progress; (2) changing social norms and attitudes about injuries and injury prevention; and (3) gaining knowledge and reducing actual injuries.

## Major accomplishments

### Creating the institutions, data systems, and an educational pipeline

Many respondents listed the creation of the National Center for Injury Prevention and Control (NCIPC) at the CDC as a major initial accomplish. Although still only receiving about 2% of the CDC budget, the very existence of NCIPC highlights the idea that injuries are a public health problem and demonstrates that our lead public health agency has made it its business to prevent unintentional injuries and violence. [Julian Waller (Waller, 1994) provides an excellent personal history of the field before the advent of NCIPC and a special issue of the *Journal of Safety Research* (Mack et al., 2012) discusses accomplishments of the NCIPC since its inception.]

Many respondents to the questionnaire listed the creation of data systems as one of the major accomplishments of the injury field. Since the mid-1980s there has been a tremendous increase in the availability of injury surveillance data—injury information collected consistently across sites and over time. CDC’s WISQARS (Web-based Injury Statistics Query and Reporting System) and WONDER (Wide-ranging On-line Data for Epidemiologic Research) interactive web-based systems allow researchers, reporters and others to immediately access disaggregate data on injury deaths. CDC’s National Violent Death Reporting System (NVDRS) provides rich circumstantial information for all suicides, homicides and unintentional firearm deaths—funds for the expansion to all 50 states were included in the most recent federal budget (the incomplete NVDRS data system has already led to scores of valuable studies of violent fatalities; a complete system will be much more useful). CDC coordinated with the Consumer Product Safety Commission to collect injury information from the National Electronic Injury Surveillance System (NEISS); this provides non-fatal data from a sample of emergency departments. Standard definitions were created for nonfatal (and fatal) injuries using International Classification of Disease (ICD) codes and most hospitals now provide information on the external cause of injury (E-codes).

Respondents also commonly cited as major accomplishments the establishment of injury researcher and practitioner organizations, primarily the Society for the Advancement of Violence and Injury Research (SAVIR), Safe States Alliance, and the Injury Control and Emergency Health Services (ICEHS). At the turn of the twenty-first century the association of injury control research centers broadened its membership to include all injury researchers, creating SAVIR; this professional organization is composed largely of academic researchers. After 1985, states and many cities created injury units in their governmental health departments, and now accredited level-1 Trauma centers are required to have injury coordinators; Safe States Alliance represents these practitioners. In the 1990s, ICEHS became a full-fledged section of the American Public Health Association (APHA), with over 650 members in 2018. The integration of researchers and practitioners, and alliances (such as those between safety people and bicycle advocates) were emphasized by some respondents as major accomplishments of the injury field.

The researcher and practitioner organizations were created, of course, because of the expanding cohort of injury professionals. The increase in injury research at academic institutions and the expanding number of practice professionals was highlighted by some respondents as major accomplishments. The integration of injury prevention into the public health school curriculum, into health departments (e.g., fall prevention programs) and the medical care system (e.g., level one trauma centers) was also noted. One respondent simply stated that the major accomplishment of the field was that “injury had established itself as a legitimate part of public health study and public health practice.”

A couple of respondents noted the establishment of field journals. *Injury Prevention* (1995), affiliated with SAVIR, provides an academic outlet for many injury scholars, and there are now a variety of journals specifically tailored for scholarly injury articles such as *Injury Epidemiology* (2014) and the *International Journal of Injury Control and Safety Promotion* (1994).

A handful of respondents noted the importance of the CDC-funded Injury Control Research Centers—for the growth of the field was part of their mission. These centers, and many other institutions, provided training for increasing numbers of new injury professionals. Many injury field accomplishments involved training, such as the Graduate Summer Session in Epidemiology at the University of Minnesota, commencing in the 1970’s and subsequently moved to the University of Michigan in the 1980’s and, during the past 20 years, the Johns-Hopkins Summer Institute in Injury Prevention and Control as well as the creation of the nine core competencies for injury prevention.

Other institutions that received mention were the World Health Organization which was instrumental in facilitating the international growth of the field, the National Institutes of Health (NIH) which provided funding (e.g. the Pediatric Trauma and Critical Illness branch of the National Institute of Child Health Development) and foundations (e.g., the California Wellness Foundation).

One respondent summed up all the many institutional accomplishments of the past 35+ years into “the very fact that there is now an injury field.”

### Changing attitudes and social norms about injury and violence prevention

Many respondents emphasized that a major accomplishment of the injury field has been a changed societal attitude about injury. More people now appear to understand that unintentional injuries are not just bad luck (e.g., “accidents”) but are foreseeable. Injuries have patterns; they are predictable and can be prevented. One respondent highlighted the policy move away from the futile ‘education alone’ approach to a Haddon-type framework that indicates the importance of changing the environment and the agent of injury.

“Violence is preventable.” Many respondents emphasized that a major accomplishment of the field has been that more policymakers have come to understand that violence is a public health issue, one that can effectively be addressed with a public health approach. Interpersonal violence—including gun violence—is not just a law enforcement problem. More police chiefs, when faced with a violent crime epidemic, now correctly state that “we just can’t arrest our way out of this problem.” Violence is a learned behavior that can be unlearned and gun violence can effectively be prevented not by solely focusing on deterring criminals, but also by changing the environment and by limiting the availability of firearms and other potential weapons. One respondent noted an increasing recognition of the role played by social determinants and some change in policy emphasis from blame to prevention.

Various respondents emphasized that a major achievement has been the promotion of the scientific approach to dealing with injury, turning away from anecdotes to data-driven identification of what is important. Another respondent highlighted the back-and-forth spread of interventions across the world, such as traffic calming, violence prevention, and alcohol strategies. Another respondent gave some credit to the injury field for the increased societal recognition of child abuse and intimate partner violence as social problems.

One respondent summed up this success in changing general attitudes and beliefs: Despite some current efforts in the United States to turn back the clock, “the prevention genie is out of the bottle.”

### Gaining knowledge and reducing actual injury

Many respondents listed the increase in knowledge about injury and violence prevention as a major accomplishment. Some gave specific examples, such as the fact that we now know that different forms of violence are interrelated, and that exposure to violence during childhood is linked to a broad range of mental and physical health problems; such evidence indicates that the prevention of violence is a lever that can impact a broad range of health problems. Another respondent thought that the documentation of impacts of interventions, such as graduated drivers’ licensure rules, impaired driving laws, and helmet laws were notable achievements. A practitioner listed the development of primary prevention strategies at the community level as a particularly important accomplishment.

Some respondents emphasized the general improvement in the science of injury control--the large increase in the number of scientific studies and the improvement in scientific methods of analysis, both leading to increased levels of useful knowledge. They also highlighted the increasing rigor of the scientific studies in the field.

Finally, many respondents listed reductions in risk factors for injury, and reductions in actual injury, as major accomplishments for the field. Risk factor achievements include the uptake in seat belt laws and seat belt use (listed by a half dozen respondents), the introduction of graduated drivers’ licenses, raising the legal drinking age to 21, the increased prevalence of smoke detectors in homes, pool fencing requirements, and legislation mandating fire-safe cigarettes. Three respondents noted improvements in technology, particularly improvements that have made cars safer in the event of a crash (e.g., airbags).

Many listed as achievements the reductions in specific injury rates, particularly in the transportation area. A half-dozen respondents mentioned motor vehicle fatalities, some specifying such areas such as reductions in child passenger deaths and alcohol-related traffic deaths. Other injury successes noted were in commercial aviation, home fire safety and child poisoning deaths.

## Major disappointments

Disappointments can be categorized into three main themes: (a) inadequate funding, (b) narrowness of the field and (c) high levels or increases in actual injuries and/or injury risk factors.

### Inadequate funding

Virtually every respondent, in one way or another mentioned funding—that the field is “not garnering resources commensurate with the problem.” The lack of funding is both a cause and effect for injury being seen a stepchild of public health—for example injury and violence prevention are still not a standard part of the curricula of all public health schools let alone in the curricula for all health professions. In many state health agencies, injury prevention has a tenuous, low impact and sometimes shrinking hold.

The lack of funding for firearms research in particular was discussed by half of the respondents with some specifically bemoaning the lack of courage on the part of the public health establishment to stand up to those who have suppressed federal support for firearms research. “Imagine where things would be with HIV (Human immunodeficiency virus), heart disease, cancer and other health problems if research had not been funded for 20 years?”

The lack of overall injury funding has been quite detrimental for the future of the entire field. We may not be recruiting new talent at a rate to sustain growth in the science and practice of the field. “Especially painful is the absence of support for training individuals to carry out injury prevention research.”

For many respondents a major cause of the inadequate funding has been the lack of good communication and advocacy by the injury field itself. There has been a failure to effectively convey the need for more research to Congress and to ensure that good studies receive greater media attention. “We still do not communicate the value of our field or the impact of our work as effectively as we need to.”

So some respondents emphasized the importance for teaching about and training for advocacy. “The rudiments of full blown, bare knuckle advocacy is nearly invisible within academia…engaging in advocacy is the necessary new frontier for reducing the horrific toll of trauma in the US and the world.” “Advocacy is the new orange.”

### Narrowness

Various respondents mentioned that the field is still too silo-ed; disciplines such as marketing, communications and behavioral economics are underrepresented. The field needs to do more to incorporate survivor advocates, plaintiff trial lawyers, advertising people, personnel of regulatory agencies, pollsters and billionaires. “We must pull in the surgeons, the rehabilitation community, laws enforcement, forensic science, and other sectors of civil society. There are no national meetings that gather all of the science leaders to discuss how we can advance the field and reduce injury and violence.”

One responder stated that work on mild traumatic brain injury was slow to develop because of limited collaborations with clinicians; efforts on opioid poisoning prevention was also a late starter because of failure to recognize the growing trend in prescription opioid use. Another respondent bemoaned the failure to address the numerous injury-related aspects of phenomena such as climate disruption due to the failure to partner across areas. The World Bank and other agencies continue to provide funds for road building and other infrastructure with little consideration for pedestrian safety. The lack of effective bridging across federal agencies seemed a concern for many who had worked in government.

There has been a failure successfully to unite occupational and non-occupational injury programs under one umbrella, or to think about transportation holistically. I personally think the narrowness extends to what we study. For example, there has been scant attention to the important role of diet or sleep in injury, the role of voluntary standards-writing organizations is not on our radar screen, and the importance of government inspectors has not been sufficiently addressed.

### Levels of injuries and risk factors

Many respondents emphasized how little we have accomplished in reducing firearm deaths. As a field, we have not been able to find successful ways to overcome the political contentiousness and deadlock on this issue.

An injury that become a severe US epidemic in the past decade is opioid poisonings. As one respondent wrote, “The major disappointment is the increase in drug deaths.” However, it was surprising how few respondents mentioned the overdose epidemic. The injury field has not yet taken a hard look at why we were so late to respond, and what we can learn from this injury epidemic that occurred on our watch.

Other injuries and risk factors that respondents pointed to as disappointments were the repeals of the 55 miles per hour speed limits and motor cycle helmet use laws, the lack of progress in window fall prevention in high-rise cities, that violence prevention is too-often ignored at the community level and the endemic levels of child abuse and gender abuse that our society tolerates.

Various respondents regretted that despite decades of efforts and some progress, too many people still believe that preventable unintentional injuries are “accidents” that are just part of the human condition.

## Advice

A final question asked for personal advice for young injury researchers and practitioners. Many of the respondents emphasized the importance of learning about and understanding the history of the field they work in. Too often young professionals make the same mistakes or end up re-inventing the wheel. Respondents emphasized that it was important to understand how the field got to where it is today, and to recognize both its accomplishments and failures.

Disappointments about the field—e.g., lack of funding, inadequate collaboration, ineffective communication—clearly influenced the advice. Should one choose this field as a profession? After discussing the lack of funding, one respondent wrote: “I could sarcastically recommend that they get a day job.” But more respondents emphasized that one should “follow your passion, not your pocketbook,” that “if you are passionate about the prevention of injury and violence, go for it—despite the odds.”

Most respondents clearly like the field they entered. “This is an amazing field to work in, both because of the wonderful people and because of the impact the work has.” Some emphasized the people: “Join the American Public Health Association (APHA) ICEHS (Injury Control & Emergency Health Services) section; the friendships and support have lasted me a lifetime and I am forever grateful.”

The importance of the work was a common theme: “This is a great field in which to be involved. There are intellectual challenges and opportunities to make a difference and change lives.” “Remember the importance of our work for people’s lives.”

The need to promote change was emphasized: “Recognize that science can only go so far, that goals require political action” and “Data without action is dead information.”

Advice on how to achieve that change was freely given: “Each piece of research must be accompanied by a communications and political agenda.” “Learn to speak about the work you do in a way that you can explain it to your grandparents and your children, and they can explain it to their friends accurately.”

Many emphasized the importance of collaboration: “Go across campus and meet with professors of business, education and others.” “Cherish and nurture the collaborative spirit—reach out and engage people even if you don’t think there is time. Think of other researchers as colleagues rather than competitors and take the time to build long term collaborations and friendships.” “Collaboration is key to success.”

Collaborating with stakeholders was seen as particularly crucial. “Engage in politics. Talk regularly with policy makers and foundations about the importance of the injury field. Be a voice—communicate frequently and often with decision-makers--and cultivate a sense of urgency.” “Incorporate the feedback of stakeholders along the way. It is of critical importance to partner with and share your research findings with grass-roots citizen activist groups whose goals are to reduce injury.” “Build support by developing community coalitions. Get out of ivory towers and make the research relevant. Financial and political support depends on making the case for how our research saves lives and reduces disability.” “Engage with diverse fields outside of public health (e.g., housing, social welfare, law enforcement) to move the needle on important public health issues—all of which have injury and violence implications.”

Persistence was highlighted: “Take a long-term perspective and don’t give up if success is not forthcoming quickly. Everything takes much longer than you hope and there will always be people and institutions fighting against you.” “The work is extremely rewarding but requires patience and persistence. Be alert. Change typically comes slowly, but be prepared to take advantage of events that provide opportunities to advance things quickly.” “Prepare for the long haul and maintain a work-life balance. You can have a career and make an impact only if you can endure the full course marathon.”

There was advice about the importance of good research: “For your work to have a lasting impact, it must be scientifically sound.” “Make sure your research is solid for it will be attacked.” “Do not undertake vast projects with half-vast ideas. Do a thorough literature review with a critical eye. Do not be afraid to criticize the work of others and accept criticism with grace.”

Many advised to “think big and be willing to take risks.” “Don’t be afraid to think and try things out of the box. Think creatively. Take risks in trying new approaches. If you are not sometimes failing, you are not trying hard enough. While old ideas and approaches are good foundations, don’t be afraid to look for new ones.”

Finally, there was advice to be fearless. “Ask the important questions. Don’t shy away from controversial topics.” “Stand up for what is right. Don’t be bullied.” “Above all preserve and uphold the value of objective science as a path to truth.”

## Conclusion

I believe it useful to occasionally step back and take stock, to think broadly and collectively about the injury field as a whole, and to share accomplishments, mistakes and misgivings. (After reading all the interesting responses to my email, I now wish I had asked more explicitly about the latter).

Much has been accomplished in the field of injury prevention in the past 35+ years. Indeed, it is difficult to believe that before the 1990s there was no federal agency focused on preventing fall injuries, drownings, sport concussions or bullying in schools. There was no WISQARS or NVDRS, no SAVIR or Safe States Alliance, no APHA injury section and few injury journals. Hardly anyone wore seat belts, and virtually no cigarettes were fire-safe. While this is an impressive list of accomplishments, the field has also had many failures and there is lots left to do.

A major problem is that injury prevention remains a stepchild in the health field, with completely inadequate funding. Injury professionals need training in advocacy to improve our standing and reduce the large injury burden in society. In addition, the injury prevention profession remains too narrow and silo-ed. We have been able to do little to reduce the level of firearm killings and we were not ready to respond effectively to the overdose epidemic that occurred on our watch.

It will be up to a new generation of injury researchers and practitioners to take the reins and lead the field. Remember that “the problems you are trying to solve involve real people who need help (but will never know that you helped them).” Be brave, be persistent and “maybe one day preventing injuries will be seen as a goal equivalent to eradicating AIDS (Acquired immunodeficiency virus) or reducing obesity.”

## Abbreviations

AIDS: Acquired immunodeficiency virus
APHA: American Public Health Association
CDC: Centers for Disease Control and Prevention
HIV: Human immunodeficiency virus
ICD: Codes International classification of diseases codes
IECHS: Injury Control and Emergency Health Services APHA section
NCIPC: National Center for Injury Prevention and Control
NEISS: National Electronic Injury Surveillance System
NIAAA: National Institute on Alcohol Abuse and Alcoholism
NIH: National Institutes of Health
NVDRS: National Violent Death Reporting System
SAVIR: Society for the Advancement of Violence and Injury Research
WISQARS: Web-based Injury Statistics Query and Reporting System
WONDER: Wide-ranging ON-line Data for Epidemiologic Research
